# University‐based academic programs in addiction studies in the regions of Australia and Aotearoa New Zealand: An overview

**DOI:** 10.1111/dar.13970

**Published:** 2024-11-03

**Authors:** Amalie Lososová, Peter Adams, Michal Miovský

**Affiliations:** ^1^ Department of Addictology, First Faculty of Medicine Charles University Czech Republic; ^2^ Centre for Addiction Research, School of Population Health The University of Auckland Auckland New Zealand; ^3^ General University Hospital in Prague Czech Republic

**Keywords:** addiction studies, Aotearoa New Zealand—Australia, degree, university education

## Abstract

**Introduction:**

Current trends in the addiction field reflect a significant emphasis on the workforce development and education. There are already some data about university‐based addiction studies programs, but not much from Australasia.

**Methods:**

The aim is to provide an overview and describe the academic programs for addiction professionals in Australia and Aotearoa NZ. The research was conducted in 2017 and updated in 2023. Firstly, university websites were searched using pre‐defined keywords, followed by a content analysis of the identified programs. The data were analysed and interpreted by using descriptive statistics.

**Results:**

We found 21 universities in Australia (13) and Aotearoa NZ (8) where 46 single programs are provided. There are three bachelor programs, nine masters, and the majority of degrees include (post)graduate certificates and diplomas. No doctorate programs are identified. The taught courses provide comprehensive coverage of the addiction field topics. Twelve programs state clearly that there is clinical practice/internship included. Application to most programs requires completion of a relevant degree and in some cases possible clinical experience.

**Discussion and Conclusions:**

In comparison to educational options in other regions, we observe a trend towards preparing university graduates for the workforce, thereby expanding the range of programs at lower levels. Most programs possibly represent clinically oriented education primarily specialising in addictions, and graduate programs in addictions for professionals with other disciplinary bases. Great emphasis is given to the quality standards of education, and also to relationship between education and labour market. Findings help opening opportunities to collaborate globally.

**Key Points**

- In total, we identified 21 universities in the regions of Australia (13) and Aotearoa New Zealand (8) where 46 various single programs in addiction studies are provided.
- Together, there are 3 bachelor's level degree programs, 9 master's programs, no doctoral degree programs; in addition, we identified 12 graduate/postgraduate certificates, 8 graduate/postgraduate diplomas; then diplomas and certificates, mostly at level 4. This appears to be a way to prepare quality and work‐ready graduates, as the spectrum of programs is focused more on the workstream at lower levels as defined by the qualification frameworks. Opposite situation is seen in Europe where the emphasis is put on the higher‐level education, as evident, for example, in provision of 18 master's programs out of 34 programs. Doctorate level of study is presumably limited to only a small group of graduates with a strong interest in research and academic careers.
- There are a high number and diversity of taught courses in the curricula, which aligns with the bio‐psycho‐socio‐spiritual model of addiction and comprehensiveness of the field. The most common includes ‘clinical assessment’ and ‘treatment interventions’, ‘research methods’, ‘public health’, ‘motivational interviewing’, ‘counseling skills’, ‘mental health (promotion)’, ‘ethical issues’, ‘biology of addiction’, ‘coexisting problems’, ‘pharmacotherapy’, ‘infant, child and youth health’.
- A key part of most addiction programs is clinical practice/internships. Two programs require students to have a placement completed before beginning study (if not working within the field already).
- In a global comparison, we need to acknowledge the broad academic spectrum and the number of universities in Australia and Aotearoa NZ providing specialised addiction studies programs. We see it as important for others around the world to find out about the differences in these educational systems. With these connections we can begin to build collaborations for the future.

## 1 INTRODUCTION

One of the main current trends in the addiction field is building partnerships with those having the same or similar interests and professional development. Central to supporting progress in the sector is having a sense of the types of professionals needed in the field, regardless whether we are talking about management and policy, services for people with substance use and/or mental health issues, or providers of necessary education and training for people who work within these services. The quality of the workforce is a critical issue for the entire addiction field and this is being similarly identified in journals [1] and also at the level of systems‐oriented foci on education and training infrastructure (e.g., WAVE project reg. no. GA‐101045870).

Currently, a considerable amount of networking is occurring within and across universities and other organisations providing training in the addiction field and this is being fostered by the International Consortium of Universities for Drug Demand Reduction (ICUDDR), a global platform for sharing knowledge, pedagogy and quality assurance in addiction studies programs. A meaningful emphasis on networking and sharing knowledge is currently being facilitated by setting international standards for addiction university education and training.

Previous research have identified more than 300 universities providing almost 400 academic addiction studies programs in the United States [2], 25 universities with more than 30 programs in Europe [3] and 6 universities with 8 programs in Africa [4]. Between October 2022 and July 2024, the number of ICUDDR member organisations from around the world (mostly consisting of university and college education providers) had increased from 316 to 452. But, there remain only two member universities from the regions of Australia and Aotearoa New Zealand (‘Aotearoa NZ’), namely the University of Auckland and the University of Melbourne. Even though we know that ‘in 2016, Australia ranked as the third largest provider of education to international students after the United States and the United Kingdom’ [5, p. 4]. There could be a host of reasons for this gap. Accordingly, we were interested in exploring what this lack in connecting could reflect in addiction university education, and then to look at ways of enabling networks to form between these regions and the rest of the world. Australia and Aotearoa NZ are both nations with highly developed and well organised systems of tertiary education that put a great emphasis on relationships between education and relevant labour markets, to a level that could be an inspiration for other countries. Although some research had been done in the area of education within this region, we could still benefit from improved insights into their education systems and especially their university programs in addictions.

Both nations in the region of Australasia (namely Australia and Aotearoa NZ) have their own specific frameworks for qualifications across their education system, including tertiary education (concretely, the Australian Qualifications Framework and the New Zealand Qualifications Framework). The main rationale for such frameworks is to ensure national recognition and consistency and also common understanding of what defines each qualification (providing clear and explicit information to both employers and prospective students about the knowledge they will acquire and what competencies they will achieve on completion of relevant qualification) [6]. The qualification frameworks in both countries provide two main pathways at the tertiary level, namely universities and vocational education and training, that provide students with two separate ways of achieving their degree or preparation either for employment or further education.

The Australian Qualifications Framework is a national education policy for regulated qualifications that comprises 10 levels (see Table 1), ranging from certificates to doctoral degrees; some qualifications are offered in more than one sector. Tertiary education is represented by vocational education and training (VET) and higher education (mainly universities). Vocational education is generally focused on practical skills and industry training, yet many VET institutions have formal pathway arrangements with universities whereby VET students have assured entry into university on successful completion of their VET qualifications [5]. Vocational education and training is provided by the TAFE (Technical and Further Education) or institutes of technology.

Higher education is offered at levels 5‐10.

**TABLE 1:** Australian Qualifications Framework based on [7].

Australian qualifications framework (AQF)			
	Tertiary education		
High school	TAFE NSW (technical and further education)	University	AQF level
		Doctoral degree	Level 10
		Masters degree	Level 9
	Graduate Diploma		Level 8
	Graduate Certificate		
	Bachelor Honours degree		
	Bachelor Degree		Level 7
	Associate Degree		Level 6
	Advanced Diploma		
	Diploma		Level 5
	Certificate IV		Level 4
	Certificate III		Level 3
Certificate II			Level 2
Certificate I			Level 1
Senior Secondary Certificates of Education			

The New Zealand Qualifications Framework (NZQF) is administered by New Zealand Qualifications Authority (NZQA) and is divided into 10 levels (see Table 2). It covers a range of qualifications from certificates to doctoral degrees. The levels are based on the complexity of learning involved. The Aotearoa NZ tertiary sector covers private training establishments, Te Pūkenga (NZ Institute of Skills and Technology; previously ‘polytechs’, and currently being replaced), wānanga (tertiary institutions mostly using Māori cultural principles), universities and workplace training [8]. There are eight government‐funded universities providing undergraduate and postgraduate degree programs, and 23 government‐funded polytechnics and institutes of technology; programs in these institutions can be both academically and vocationally focused [9].

**TABLE 2:** Aotearoa NZ Qualifications Framework, based on [6].

New Zealand qualifications framework (NZQF)			
	Tertiary education		
Secondary school	Institutes of Technology and Polytechnics	Universities	NZQF level
		Doctoral degree	Level 10 (3 years)
	Master's degree		Level 9 (1, 5–2 years)
	Postgraduate Diploma		Level 8 (1 year)
	Postgraduate Certificate		
	Bachelor Honours degree		
	Bachelor degree		Level 7 (3 years)
	Diploma		Level 6 (1 year)
			Level 5 (1 year)
	Certificate		Level 4 (3–9 months)
NCEA			Level 3
NCEA			Level 2
NCEA			Level 1

Abbreviation: NCEA, National Certificate of Educational Achievement.

It is important to mention that in both Aotearoa NZ and Australia, the terms for tertiary education levels can be perceived differently within these countries. For example, in Australasian regions ‘graduate’ commonly means the level in between undergraduate and postgraduate, and ‘postgraduate’ often represents a masters level program.

This paper focuses specifically on alcohol and drug/addiction university education in Australia and Aotearoa NZ, and adopts a comprehensive research used previously in mapping addiction studies programs at universities around the world. The main aim of this survey is to identify the programs and describe them according to their specific characteristics with an ambition of fostering collaboration between universities and of helping to improve the quality of addiction education globally.

## 2 METHODS

The aim of this study is to provide an overview of academic programs for addiction professionals in Australia and Aotearoa New Zealand. In doing so, we sought to find out in which universities they are occurring, how their coverage differs between regions and what are the unique characteristics of each program. The research was originally conducted in 2017 and updated in 2023. We focused on information provided on organisational websites. The main focus was on university‐based degree and certificate education programs, using a few pre‐defined keywords: ‘addiction studies’, ‘drug & alcohol studies’, ‘master in addiction’, ‘addiction counseling’, ‘substance use’ + combination of above mentioned words with ‘New Zealand/Australia’.

The term ‘addiction studies’ refers to any university‐based program that is either primarily focused on education in the addiction field, or is connected with treatment, prevention, research, or policy related to substance use and addictive behaviours. The basic inclusion criteria were, first, having website information about the program in the English language and, second, providing university‐based programs that were defined as those awarding a degree, a diploma or a certificate. Non‐university‐based programs, continuing professional development and education, and summer school programs were excluded regardless of whether they were offered by a university. We also did not reviewed programs offered as vocational education and training if not provided by a university.

The research was conducted in two phases. The first phase consisted of the internet search of programs in addiction studies according to the above‐mentioned keywords. The second phase consisted of a content analysis of the identified academic programs guided by the following questions: (i) What university‐based study programs in the addictions field exist in Australia and Aotearoa NZ?; (ii) What degrees or other awards can be obtained?; (iii) What professional backgrounds do the programs represent (e.g., medical, social, psychological or other)?; (iv) What are the major themes of these programs, as reflected in the program titles?; (v) What are the common methods of delivery and durations of the study programs?; (vi) What are the entrance requirements and fees?; (vii) Is clinical practice a part of the study programs?; (viii) What courses are taught in addiction studies programs?

The data were analysed and findings compilated by using descriptive statistics in Microsoft Excel.

## 3 RESULTS

In total, we found 21 universities in Australia (13) and Aotearoa NZ (8) in which 46 single programs in addiction studies are provided. From the Australian study programs, one in addiction medicine is not a separate clearly defined (and accredited) study program in fact, the topic of addiction is generally integrated into teaching of medical students.

Within the searched period of time (2017 and 2023), we identified changes in provision of programs. Two programs in addiction studies have been replaced by the general program without an addiction stream. Nine programs are no longer available (specifically two master's, one bachelor's program, and the rest are various certificates or diplomas). Three programs at one university were not offered in the academic year 2022 but are still operated.

### 3.1 Australia

In Australia we found 13 universities (out of in total 41 local universities in Australia, both public and private; [10]) that provide in total 24 addiction study programs (for detailed information see Table 3). Ten programs specify information about their institutional platforms (i.e., faculties or departments where they are operated), of these five programs are rooted primarily in the platform of medical disciplines (nursing, medicine or health sciences faculties), and, in three cases, specifically focused on public health. Two programs are offered in a school of social work.

**TABLE 3:** Universities, study programs and degrees available in Australian region and their form and length.

University	Study program	Degree	Form and length (in years)
Edith Cowan University	Psychology and Addiction Studies^a^	Bachelor of Arts	Full‐time (3) Part‐time (6)
The University of Adelaide	Addiction Studies	Master of Science	Full‐time (1) Part‐time (2)
	International Addiction Studies	Graduate Certificate	Full‐time (1)
	International Addiction Studies	Graduate Diploma	Full‐time (2)
	Alcohol and Drug Studies	Graduate Certificate	Full‐time (6 months)
	Addiction and Mental Health	Graduate Diploma	Full‐time (1)
The University of Notre Dame	Substance Use Nursing^b^	Graduate Certificate	Full‐time (6 months)
The University of Newcastle	Alcohol and Other Drugs	Master of Health Science (specialisation)	Full‐time (x) Part‐time (x)
University of Southern Queensland	Counselling (Alcohol and Drug Studies)	Graduate Certificate	Part‐time (1)
	Counselling (Alcohol and Drug Studies)	Graduate Diploma	Full‐time (1) Part‐time (2)
	Counselling (Alcohol and Drug Studies)	Masters	Full‐time (2) Part‐time (4)
The University of Queensland	Alcohol, Tobacco and Other Drug Studies^c^	Graduate Certificate	Part‐time (1)
	Alcohol, Tobacco and Other Drug Studies^b^	Single major program	x
Monash University	Addictive Behaviours	Masters	Part‐time (3)
	Addictive Behaviours	Graduate Diploma	x
	Addictive Behaviours	Graduate Certificate	x
The University of Sydney	Indigenous Health (Substance Use)^b^	Masters	Full‐time (1)
	Addiction Medicine	x	x
Charles Sturt University	Social Science (Addiction Studies)^b^	Masters	x
Australian College of Nursing	Drug and Alcohol Nursing	Graduate Certificate	x
RMIT University	Alcohol and Other Drugs	Certificate	Full‐time (1)
	Alcohol and Other Drugs	Diploma	Full‐time (1)
University of Tasmania	Addiction Studies for Health Professionals	Graduate Certificate	Part‐time (1)
The Royal Australasian College of Physicians	Addiction Medicine	Advanced training program	Full‐time (3)

^a^ This program no longer exists in 2022, and has been replaced with another one – general Bachelor of Psychology, without an addiction stream.

^b^ This program no longer exists in 2022.

^c^ This program no longer exists in 2022, and has been replaced by the Graduate Certificate in Public Health, without an addiction stream.

**FIGURE 1:**
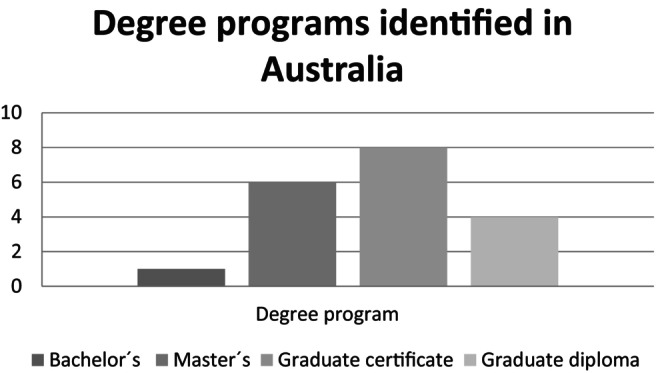
The number of addiction studies programs identified in Australia, according to the degree.

One program is being considered as provided at undergraduate level and eight at each, graduate and postgraduate levels. Specifically, 19 degree programs are shown in Figure 1. The remainder of the programs identified include one major program (a part of the degree program which consists of a specified group of courses in a particular discipline or field), one national certificate (level 4), one diploma program and one training program (in medicine).

The most common key words in program titles include ‘alcohol and drug (studies)’ (10×), ‘addiction’ was mentioned four times separately and six more times in a combination ‘addiction studies’. The term ‘counseling’ was mentioned three times in the program title. The remaining key words include ‘nursing’, ‘substance use’, ‘health’, ‘medicine’, then ‘social studies’, ‘mental health’ and ‘psychology’.

Clear educational continuity (i.e., the opportunity to get education in a chosen discipline, here addiction studies, at various education levels, for example a bachelor's degree followed by a master's degree or a certificate followed by a diploma in the same topic) is guaranteed in 12 programs, either at a previous or higher level.

There is a diversity in the offerings of courses available, both in Australia and Aotearoa NZ. The most common courses in both regions are presented in Table 4. An interesting model at Monash University involves students having the option to choose between two streams or specialisations, respectively a coursework stream for clinical work or a research stream.

**TABLE 4:** Number of repetitions of courses taught at Australian and Aotearoa New Zealand universities within addiction studies programs.

Australia		Aotearoa New Zealand	
Course title	Number	Course title	Number
Research methods; research projects	9	Assessment and intervention	16
Clinical assessment	8	Professional practice in addictions/addiction practicum	10
Treatment interventions	8	Coexisting problems	9
Public health	7	Alcohol, tobacco and other drug studies	7
Introduction to addiction	6	Theory and skills in counselling practice	7
Motivational interviewing	6	Pharmacotherapy	7
Counselling skills	6	Introduction to addiction studies	6
Clinical practicum	6	Working with diversity	6
Ethical issues	5	Mental health (promotion)	6
Biology of addiction	5	Motivational interviewing	5
Supervision	5	Research methods	5
Psychoactive drugs	4	Infant, child and youth health	5
Epidemiology	3	Criminal justice and law	4
Health promotion	1	Gambling	4
Pharmacotherapy	3	Client centred practice skills	3
Relapse prevention	2	Social problems	3
Psychology	1	Pharmacology	3
		Tobacco control	3
		Group work	3
		Cognitive‐behavioural therapy	3
		Psychology	2
		Ethics	1
		Biology of addiction	1
		Addictive consumptions and public health	1
		Nursing	1
		Prevention	1
		Case management	1
		Organisation of health systems	1
		Māori and Pacific health	1

The most common entry requirement for addiction studies programs is a relevant previous degree (11 programs), 3 other programs add the condition of previous professional work experience, sometimes specifying the extent expected. Two programs require applicants to provide proof of proficiency in English (as being available also for students from countries with other language). Two programs invite the applicants to a personal interview, another two have no requirements and one program requires a minimum level of the student's grade‐point average, and one asks for an appropriate referee's endorsement.

Seven programs have no information about the fees, the remaining 15 state that students are required to pay fees for studying. One program specifies the amount as AUD 19,000 (USD 13,000) per year.

Ten programs state that clinical practice in some form is part of their curriculum. Seven programs state they provide clinical practice opportunities for students. One has two practicums in the third year of study, two specify 100 hours of practice and active role of the student in negotiating the supervised placement. One program declares there is no practicum in the curriculum.

The most common assessment techniques for checking on the academic achievement includes written exams or essays (8×), discussions (5×), supervised exams, assignments (4×), (online) self‐assessments or presentations or attendance in class (3×). Other less common forms include theses, knowledge tests, case studies, portfolios, research projects, role‐plays and practice reports.

### 3.2 Aotearoa New Zealand

In the region of Aotearoa NZ, eight universities were identified as offering 22 study programs in addictions (detailed information in Table 5). Nine programs are offered at medically oriented schools (‘medical and health sciences’, ‘population health’ or ‘psychological medicine’) and five operate at schools of health and social sciences. The remainder programs present no information about the faculty or department where they are offered.

**TABLE 5:** Universities, study programs and degrees available in region of Aotearoa New Zealand and their form and length.

University	Study program	Degree	Form and length (in years)
The University of Auckland	Alcohol and Drug Studies	Postgraduate Certificate in Health Science	Part‐time (1–2)
	Alcohol and Drug Studies	Postgraduate Diploma in Health Science	Full‐time (1) Part‐time (4)
	Addiction Studies	Master of Health Practice	Full‐time (x) Part‐time (x)
Auckland University of Technology	Mental Health and Addictions	Postgraduate Certificate in Health Science	x
	Mental Health and Addictions	Postgraduate Diploma in Health Science	x
	Mental Health and Addictions	Master of Health Practice (MHPrac)	x
	Addictions Pathway^a^	Postgraduate Certificate in Health Science	Full‐time (6 months) Part‐time (x)
	Addictions Pathway^a^	Postgraduate Diploma in Health Science	Full‐time (1) Part‐time (x)
Wellington Institute of Technology	Addiction Studies^b^	Bachelor	Full‐time (3) Part‐time (6)
	Applied Addiction Studies^b^	Graduate Diploma	Full‐time (1) Part‐time (2)
	Addiction, Alcohol and Drug Studies^b^	Graduate Diploma	Full‐time (1) Part‐time (2)
	Alcohol and Drug Studies^a^	Diploma	Part‐time (2)
	Alcohol and Drug Studies (Support Work)^a^	Certificate	Part‐time (1)
Southern Institute of Technology	Mental Health and Addiction Support	Certificate (Level 4)	Full‐time (1)
Unitec Institute of Technology	Health and Social Development (Youth Addiction Support)^a^	Bachelor	Full‐time (3) Part‐time (x)
	Mental Health and Addiction Support	Certificate in Health and Wellbeing (Social and Community Services) (Level 4)	Full‐time (40 weeks)
NorthTec	Mental Health and Addictions	Certificate (Level 4)	Full‐time (1 semester)
Open Polytechnic (Kuratini Tuwhera)	Mental Health and Addiction Support	Certificate (Level 4)	Part‐time (18 months)
University of Otago	Health Sciences (Addiction and Coexisting Disorders)	Postgraduate Certificate	x
	Health Sciences (Addiction and Coexisting Disorders)	Postgraduate Diploma	x
	Nursing (Clinical) Addiction Pathway	Master of Health Science	x
Otago Polytechnic	Te Taketake Diploma in Applied Addictions Counselling	Diploma (Level 7 = equivalent of the final year of a university degree.)	x (2)

^a^ This program no longer exists in 2022.

^b^ This course is not currently available in 2022.

**FIGURE 2:**
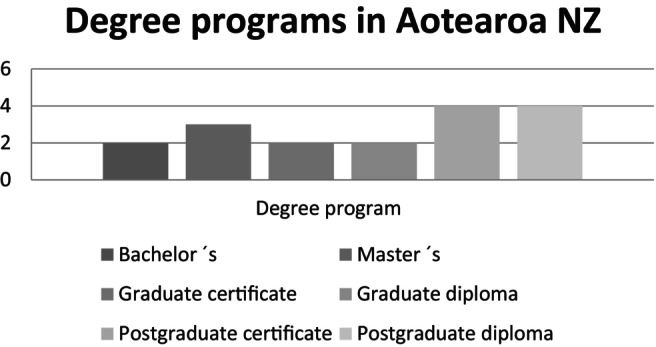
Number of the most common degree programs in addiction studies provided in universities in Aotearoa New Zealand.

Two programs are described as undergraduate (bachelor's), two as graduate and 11 as postgraduate level programs. There are two bachelor's programs, three master's programs (Master of Health Practice; Master of Health Sciences) and no doctoral programs in addictions. Most programs are offered as certificates or diplomas (see Figure 2). Out of 22 programs, 7 provide no information about the study level and study form on their websites.

The length of study differs according to the degree level and form of study, see Table 5.

We identified key words and themes included in study program titles, both used separately or combined. The most frequent term is ‘addiction’ (18×) following by ‘studies’ (8×), ‘mental health’ (7×), ‘drug’, ‘alcohol’ and ‘support’ (5×), ‘health’ (3×), ‘coexisting disorders’ and ‘science’ (2×). The most often combinations are ‘mental health and addictions’ (7×), ‘alcohol and drug studies’ (5×) and ‘addiction studies’ (3×).

Continuity in education is ensured in 15 programs, most often in the form of complementary certificates, diplomas, or follow‐on master's degrees. Four programs state there is no continuity.

The requirements for admission most often include a bachelor's degree or other qualification plus 2‐3 years of work experience (nine programs), so the program aims at reaching those who are already employed in the field. Seven programs require evidence of proficiency in English (e.g., International English Language Testing System exam [IELTS], or Test of English as a Foreign Language exam at defined levels [TOEFL]), as being open both to domestic and international students whose language is other than English. Seven require adequate grade‐point average credits or achievement of the National Certificate of Educational Achievement Level 3 (this is an official secondary school qualification in Aotearoa NZ). In five cases, the university requires current or previous clinical work experience or voluntary clinical placement in the addiction field for a defined length; or they invite the applicants for an interview. Three programs expect applicants to deliver an appropriate external reference.

Eighteen programs in Aotearoa NZ indicate they charge fees. Two programs specify an amount of NZD 6000 (USD 3500) or NZD 10,000 (USD 6000) per year for domestic students (but with fees differing for the international students). Two programs offer the funding option by government through a mental health workforce agency, Te Pou. One program is completely free and three programs have no information about fees.

Five programs state they provide clinical practice, often required in each semester or academic year. The extent differs from 50 150 hours or 400 hours for 2 years of study. Two programs require students to have a placement completed before beginning study (if not working within the field already). But no details on this are available, including the number of hours dedicated specifically to clinical work in prevention, treatment, harm reduction or recovery. The remainder do not provide information about clinical practice within the curricula.

The list of most frequent courses with the numbers of repetitions is shown in Table 4.

As for assessment methods, mostly program websites provide little information. Only two specific forms of assessments are mentioned that involve workplace‐based tasks verified by a supervisor and workbook assessments. Students presumably receive more information once enrolled.

On the basis of the titles and other characteristics, we attempted to diversify the programs as suggested in the typology by Miovský et al. [11]. We were unable to say definitively whether the programs are clinically, research or theoretically oriented, nor could we discern whether they focused specifically on particular aspects of addiction (such as prevention, treatment or law). Nonetheless, we see 12 programs stating that clinical practice or internships are a key part of their studies, and when we consider the courses taught, we notice a meaningful representation of clinically oriented courses, such as ‘assessment and intervention’, ‘professional practice in addictions/addiction practicum’, ‘theory and skills in counselling practice’. Three universities in Aotearoa NZ include clinical supervision for their students, another three offer an opportunity for being registered as drug and alcohol practitioners when completing specific courses within postgraduate certificate/diploma in health sciences and achieving an applied health or social degree (in Addiction Practitioners' Association Aotearoa New Zealand; DAPAANZ). Two programs specifically mention in the title that they are ‘applied’ which we assume means clinically oriented. In Australia, Monash University even have two different streams for studies of the ‘Addictive Behaviours’ programs, a separate stream for clinical practice and a specific research stream.

## 4 DISCUSSION

This paper is focused on university‐based tertiary education programs in addiction studies in Australia and Aotearoa NZ. Below we discuss key findings and related backgrounds, in general and in comparison with identified educational options within other world regions and also with reference to a typology proposed by Miovský et al. [11]. In considering ways in which programs might be strengthened, the following also discusses its relevance to national and regional contexts in relation to formulating quality standards, particularly regarding the intersection between education and labour markets. Last but not least, we consider global opportunities for universities to interconnect and collaborate.

### 4.1 University‐based addiction studies: Context and global comparison

In the addiction field over recent decades, there has been a greater focus on workforce development and on the provision of formal qualifications [12]. The growing emphasis by employers in requiring professionally defined minimal qualifications [13, 14] has also created an imperative for the provision of appropriate qualifications to meet this demand for increased professionalism and elevated skill levels [15]. We view these trends as positive but also acknowledge that it can have negative cost implications for employers, an issue that is rarely openly discussed. Possible answer to this issue could be seen in providing various levels of training, with various skills and competencies gained, and appropriate career ladder in the country (the higher and more specialised the position and competencies, the higher education required).

To become an addiction professional in Aotearoa NZ, three positions can be chosen: Addiction Support Worker, Addiction Peer Support Worker (both require certificate at NZQA Level 4) or Addiction Practitioner/Health Professional. Addiction practitioners are usually health professionals who are qualified and competent to independently provide addiction interventions that include comprehensive assessment and treatment planning and delivery [16]. What is required for this position is both an undergraduate health qualification, namely at a bachelor level, and professional registration with DAPAANZ (the practitioner registering body; [17]). DAPAANZ also evaluates programs and registers them according to their alignment with their national practitioner competencies.

Accordingly, there is a diversification of vocational education which caters largely for those who have a professional interest in this area but few formal qualifications; and the higher education at universities. The VET sector in Australia has, accordingly, developed a suite of courses for those interested in the addictions field. The National Centre for Education and Training on Addiction (NCETA) in Australia previously undertook a review of available alcohol and drug and mental health courses in higher education and VET sectors [18]. Then, Roche and White [15] conducted a survey, similar to what is presented here, yet focused specifically on training providers of alcohol and drug related VET qualifications. There is a wide range of registered training organisations that span schools to universities and include public and private institutions. A total of 69 training providers were identified who offer one or more accredited courses. Of these, 63 training providers offer a Certificate IV (level 4 qualification bringing advanced skills and knowledge) in Alcohol and Other Drugs Work, 35 offer a Diploma of Community Services (Alcohol and Other Drugs) and 31 offer a Diploma of Community Service (Alcohol, Other Drugs and Mental Health). To compare, there are 21 universities providing 46 addiction‐focused specialised study programs identified in both regions. The numbers of specialised addiction training programs might reflect the realities of the region, the needs of practice and the career hierarchies in the field.

Together, there are three bachelor's level degree programs, nine master's programs and no doctoral degree programs. In addition we identified 12 graduate/postgraduate certificates, 8 graduate/postgraduate diplomas (on the same NZQA Level 8 but differently named); then diplomas and certificates, mostly at Level 4. This appears to be a way to prepare quality and work‐ready graduates, as the spectrum of programs is focused more on the workstream at lower levels as defined by the qualification frameworks. A similar system of educational options and qualification frameworks as in Australia and Aotearoa NZ exists in the United Kingdom and in the United States [2] where highly variable educational systems are in place that also have lower‐level qualifications available (these are called ‘foundation degrees’ in the United Kingdom, or ‘associate degrees’ in the United States). These undergraduate qualifications are the most common (53% of 392 programs in the United States), and mostly serve to directly prepare students for employment or their next level of study [19]. Opposite situation is seen in Europe where the emphasis is given on higher level education, as evident, for example, in the offering of 18 master's programs out of 34 programs, and also not having developed the system of lower‐qualification educational options [3].

Moreover, we identified no specific doctoral programs in addictions in Australia or Aotearoa NZ. For students interested in further study after completing their master's, Monash University in Melbourne offers optional PhD level study with a possible focus on addictions and addictive behaviour. We had previously identified four PhD programs in Europe [3], five PhD programs in the United States [2] and no PhD programs in Africa [4]. Doctorate level of study is presumably limited to only a small group of graduates with a strong interest in research and an academic career.

Globally, it seems that the current educational trend is in moving toward more practical and ready‐to‐work education and a constantly growing number of programs at lower levels, that is, short‐term and narrowly focused specialisations. Yet, both processes preparing ready‐to‐work employees in lower levels of education, and requiring higher qualifications for specialised and highly skilled positions and leadership, are present and needed.

A sizeable number of Australian and Aotearoa NZ programs (14 of 26 programs that stated this information; 53.8%) are offered on medical or health sciences schools or faculties. In comparison to other regions, we have 11.8% of such programs in Europe [3]; and 66.7% in Africa [4]. This might suggest any preference in particular approaches to addiction, however, instead, all programs provide coverage of issues represented by the bio‐psycho‐socio‐spiritual model of addiction [20]. Yet, there are various reasons for choosing medical or health faculties for addiction studies programs. First, is the input from supportive staff in the faculty, or secondly, the content of a program can over time become appropriately embedded in the educational systems of the faculty. Or, thirdly, this finding might reflect the legal framework and the associated concept of the addiction professional in the respective region. In Australia, an addiction practitioner is considered a health professional, accordingly, 8 of 10 programs which state which faculty they are in, are offered at the medically oriented ones. This is not necessarily the case in other regions where addiction specialists are considered more as social science professionals, partially depending on the faculty operating the program. Accordingly, overall, substance use disorders and addictions are seen as important health issues and it appears reasonable for them to be dealt with as health issues by health care professionals.

We also note a specific emphasis on addiction and mental health in Aotearoa NZ university programs. Most of them seek to integrate mental health and addiction issues. Historically in Aotearoa NZ, addiction services were dominated by psychiatrists and medical doctors, but later other professions (such as psychologists and social workers) became prominent and slowly the notion of addiction professionals began to emerge. Accordingly, the collaboration of addiction medicine specialists with those from other disciplinary bases has a visible presence in addiction services. Although addiction medicine is taught separately, being strictly a domain of specialised medical colleges, it is an undoubtable part of the addiction discipline and education. The specialty field of addiction medicine was approved in 2009 by the Australian Government, then the Royal Australasian College of Physicians, namely the Chapter of Addiction Medicine [21, 22].

In Australasia, all students are required to pay at least some of the costs of their program but only three programs state the total amount. This may be partially explained by the high variability in what the fee is based on. It can be in a form of total study fee, or per‐points fee, or, most commonly, fees related to the actual courses the student is taking. So, the amount of money spent on studies is dependent on the actual enrolment of each student and this is not easily stated. Nonetheless, this tendency not to state enrolment costs reflects the common realities in other regions [2, 4].

The COVID 19 epidemic affected people's lives in so many ways across the world and this included a move to online training options. Online opportunities bring addiction education closer to people in different countries, or lacking the time or money to study face‐to‐face [23]. In Australia, four programs are offered both face‐to‐face and online, another four programs are offered only online and one program in Aotearoa NZ is offered in a distance learning format. It is seen as important for universities and addiction studies programs, and also for clinical services [24], to respond to current trends and be open to adapting to new methods of teaching, and yet, however, face‐to‐face teaching still remains far superior especially for practice‐oriented training.

In Australian university addiction programs, the majority include courses focused on research. This is similar to what is seen in Europe and Africa [3, 4]. The programs focus on addiction studies graduates becoming conversant with research methods and understanding the meaning of evaluation and other methodologies used in the addiction field. Of course many courses are focused specifically on addiction topics or on related disciplines and techniques used in prevention, treatment, harm reduction and public health, however, curricula across the world highlight a strong commitment to transdisciplinary approaches to addictions. In spite of this positive finding, there remain important and not inconsiderable gaps within the courses. We see a vast majority of courses focused on treatment and clinical practice, and oppositely relatively low proportion of prevention, and even less focus is given on harm reduction topics. The same proportion is seen in European addiction degree programs [3]. This issue highlights the need to maintain continuing research focus on AOD field needs and addiction workforce characteristics. It also might be explained by the regional differences, as Australia and Aotearoa NZ and Europe do not mostly use Universal Prevention/Treatment Curricula, creating their own study programs and systems. As presented by the ICUDDR member universities [25], where a majority is consisted of Asian and African universities, there is a balance between programs treatment‐ and prevention‐oriented as well as support in providing addiction education based on the universal curricula.

Considering the descriptions of the target study groups, the Australasian programs appeared more focused on a specialisation following education in other general disciplines such as nursing or social work. For example, graduate certificate programs in ‘Addiction Studies’ or ‘Alcohol and Drug Studies’ at the University of Adelaide or programs at the Monash University are described as beneficial in a range of professions that interact with drug and alcohol addiction in health and social welfare sectors, and related areas such as government or policy, who seek to broaden their knowledge and skills in addiction field. These might include medical practitioners, nurses, psychologists, social workers, pharmacists, educators, law enforcement officers, counsellors, and project and policy officers. Within the results we identified programs named ‘Addiction Medicine’, ‘Psychology and Addiction Studies’, ‘Drug and Alcohol Nursing’ or ‘Social Science (Addiction Studies)’ that could also serve as the examples of such specialisations. Most programs in these regions probably represent Type 1, 2 or 3 according to the proposed matrix of education made by Miovský et al. [11], that is, clinically oriented both in undergraduate and graduate programs primarily specialising in addictions, and graduate programs in addictions for professionals with other disciplinary bases.

In the eight Aotearoa NZ universities, where clinical experience in the addiction field is a requirement for entry, regardless the level of the degree program, the assumption is that this prior involvement signals a deeper interest in the field. On the other hand, this requirement is unusual given the way study in medicine or nursing or psychology focuses on criteria other than prior experience, as, to some extent, this expectation could act as a barrier for suitable people to enter the field. In (addiction) medicine generally or addiction studies in other regions, the process is usually the opposite. Undergoing a robust training and internships within and after the studies precedes entering the clinical practice, as is functioning, for example, in the form of residential fellowship trainings in medicine [26].

### 4.2 Relationship between tertiary education and labour market

In Australia, a great emphasis is put on the graduates' skills and on their future employability, regardless of whether they are national or international. Combined with strong economy and low unemployment rates, Australia offers strong employment outcomes for graduates [27]. Also, labour‐market outcomes by education level reflect well on the Australian education system. For example, 91.7% of domestic graduates in undergraduate education were employed full‐time 3 years after completion of their course in 2023, when compared to 2020 data, we see that 70.3% of graduates were employed in four to six months after course completion. For graduates of postgraduate programs, the employability rate is even higher, 86% in short‐term and 94.8% in medium‐term after completion. The employability rate is generally high in graduates in rehabilitation, medicine and psychology [28], where addiction specialisation could be also included. The international graduates who create a meaningful proportion of all students we see the success in full‐time employment as 45.9% in short‐term and 85.3% in medium‐term for undergraduate programs, and 49.6% in short‐term and 87.3% in medium‐term in postgraduate programs graduates [28]. To continue this growth in the sector, the Australian Government's National Strategy for International Education 2021–2030 [29] speaks to the soft‐power importance of international education cultivated through two‐way mobility programs for both students and researchers and to the way qualification recognition facilitates global workforce mobility [5].

It is particularly noteworthy that the majority of both early‐ and mid‐late career workers in Australia have a general tertiary qualification [30], perhaps indicating the growing professionalism of the sector [31, 32]. This encouraging finding also suggests that workers with different skill sets, backgrounds and aspirations may be increasingly attracted to, or actively recruited into, the addiction field; or could reflect a requirement of programs that the applicants are already employed in the field while motivated to increase their education. Additionally, workers with graduate‐level qualifications are well placed to undertake advanced training and acquire new skills, compared to workers who enter the sector with more limited or basic qualifications. A ‘one size fits all’ reductionist approach to workforce development is insufficient in ensuring that early career workers are appropriately trained and supported and in safeguarding the sustainability of the addiction workforce to meet increasingly complex demands.

Roche, Skinner and McEntee [30] focused on a very important group of workers, those who are at the beginning of their career in the addiction field (having limited practical experience, up to 3 years), including recent graduates. Appropriate professional development strategies play a critical role in building capability in the workforce, especially with new workforce members. However, for workers in the addiction field overall, regardless of their years of experience or sector of employment, the top priority for professional development identified were advanced clinical skills and clients with complex needs. This finding underscores a pervasive lack of focus on advanced skill development in spite of increasing complexity in addiction presentation, and so brings important feedback to the education providers and employers about what is needed in training. It also illustrates the need for graduates and workers to be involved in discussions regarding education and practice requirements. To fill this information gap, White (2023) conducted a grounded theory study of experiences and views of postgraduate students of training programs in Aotearoa NZ. Her work highlighted important aspects about strengths and limitations of the current systems and what might be done to improve the current system of workforce preparation [33]. In Aotearoa NZ, Adams et al. [34] claimed ‘little could have been achieved without ongoing cooperation between education providers, government agencies, and addiction services’ to improve both the content of education and the standards of practice.

Moreover, continuous evaluation and feedback provides information about the weaknesses and challenges for the future. One such challenge is bridging the gaps in education availability—both geographically and demographically. In the case of Aotearoa NZ, considerable effort is going into adjusting education and addiction studies programs to meet the needs of Maori (indigenous) and Pacific and Asian cultures. Their needs are of increasing importance so interest in addiction education is growing as is their success in gaining higher qualifications including tertiary education [35].

In these regions, there have been visible attempts by addiction professionals to have a bigger say in negotiating their terms and conditions of employment. Two examples in Aotearoa NZ are represented by the DAPAANZ which represents the professional interests of the addiction workforce; and Te Pou, a national centre for workforce and leadership development for the addiction workforce.

### 4.3 Quality of addiction education and interconnections

The emphasis on quality and appropriate standards in addiction education is currently a high priority topic worldwide. In Australia and Aotearoa NZ government agencies have developed a set of standards for university education as well as a set of assessment processes. On top of having a well‐developed standardisation process, a great strength of the Australian education system lies in its robust regulatory framework and transparent accountability mechanisms. There are two main regulatory and quality assurance bodies relating to higher education and VET institutions: the Tertiary Education Quality and Standards Agency (TEQSA) for higher education, and the Australian Skills Quality Authority (ASQA) for VET education. These government organisations are responsible for registration/re‐registration of institutions and accreditation/re‐accreditation of programs, and also for assessing the performance of higher education providers against the Higher Education Standards Framework, respectively National Standards Framework, and undertakes both compliance and quality assessments of providers [36].

Under a Aotearoa NZ legislation, the New Zealand Vice‐Chancellors' Committee (operating as Universities New Zealand—Te Pōkai Tara) exercises the powers of program approval and accreditation held by the NZQA for the rest of the tertiary education sector. Universities New Zealand has delegated its powers to the Committee on University Academic Programs (CUAP), which undertakes its program approval and accreditation functions within policies such as the gazetted criteria for program approval developed by NZQA after consulting the university sector. Another quality assurance organisation is the Academic Quality Agency for New Zealand Universities (AQA) that encompass both academic quality assurance and academic quality enhancement, including the dissemination of good practice [37].

Similarly, other countries prepare their requirements and standards, yet we are also witnessing significant attempts to prepare international standards for university‐based programs in addiction studies by the National Addiction Studies Accreditation Commission (NASAC and ICUDDR, [38]) so as to set minimal standards for skills and knowledge of addiction workforce internationally. This process could bring an important step for universities and training and education providers to connect or even to collaborate and thereby strengthen their position.

Universities Australia is a peak body for the tertiary education sector that advocates for the social, economic and cultural value of higher education and research as well as providing expert policy advice, analysis and statistical evidence, and media commentary on higher education. They also make submissions, develop policy across the sector, represent the universities on government and industry‐appointed bodies and partner with the university sector in other countries to enable bilateral and global collaborations. The member universities also have access to collective procurement and licensing agreements [39]. A similar association is functioning in Aotearoa NZ, the Universities New Zealand—Te Pōkai Tara, which umbrellas all eight universities in the country. Similarly, ICUDDR represents such umbrella and advocacy for universities and training providers from the whole world.

### 4.4 Limitations

As a first attempt to comprehensively map university addiction education in the world, this study has several limitations. English was the only language used and this could have excluded some programs from the search. Some universities had only limited information about the study programs publicly available on their websites. Another limitation is the internet search method that was used. It is an important source of data but the information available varies in terms of its quality and relevance. To minimise this bias, we only used official university websites. The search is only based on certain key terms so it is therefore possible that there are still some meaningful key words and terms missing. There could be other programs focused on addiction‐related topics that we were not able to include.

## 5 CONCLUSION

The paper aims at presenting an overview of available university‐based addiction study programs in Australia and Aotearoa New Zealand, being an important sector for preparation and development of addiction professional workforce. It brings detail characteristics of identified programs and relates the results to the broader context of the regional educational system, career ladder and quality assurance.

In a global comparison, we need to acknowledge the broad academic spectrum and the number of universities in Australia and Aotearoa NZ providing specialised addiction studies programs. We see it as important for others around the world to find out about the differences in these educational systems and share the inspirational and innovative ways of workforce development. There are also many opportunities for future research that flow from this one presented, such as a more detailed comparison of existing programs or compiling of case studies of best practice in addiction studies programs that could provide some guidance and inspiration for those looking to develop similar programs.

Alongside the above mentioned opportunities, the results enable the world and Australasia to connect and begin to build collaborations for the future.

## AUTHOR CONTRIBUTIONS

Each author certifies that their contribution to this work meets the standards of the International Committee of Medical Journal Editors.

## CONFLICT OF INTEREST STATEMENT

The authors report there are no competing interests to declare. The authors alone are responsible for the content and writing of this article.
